# Consumption of water containing a high concentration of molecular hydrogen reduces oxidative stress and disease activity in patients with rheumatoid arthritis: an open-label pilot study

**DOI:** 10.1186/2045-9912-2-27

**Published:** 2012-10-02

**Authors:** Toru Ishibashi, Bunpei Sato, Mariko Rikitake, Tomoki Seo, Ryosuke Kurokawa, Yuichi Hara, Yuji Naritomi, Hiroshi Hara, Tetsuhiko Nagao

**Affiliations:** 1 Haradoi Hospital, Department of Rheumatology and Orthopaedic Surgery, 6-40-8 Aoba, Higashi-ku, Fukuoka 813-8588, Japan; 2 MiZ Company, 16-5 Zengyo 1-chome, Fujisawa, Kanagawa, 251-0871, Japan; 3 Midorino Clinic, 7-26-1 Aoba, Higashi-ku, Fukuoka, 813-0025, Japan

**Keywords:** Arthritis, Rheumatoid, Oxidative stress, Reactive oxygen species, Molecular hydrogen, 8-hydroxylguanine, Hydroxyl radical: DNA repair, Error protein

## Abstract

**Background:**

Rheumatoid arthritis (RA) is a chronic inflammatory disease characterized by the destruction of bone and cartilage. Although its etiology is unknown, the hydroxyl radical has been suggested to be involved in the pathogenesis of RA. Recently, molecular hydrogen (H_2_) was demonstrated to be a selective scavenger for the hydroxyl radical. Also, the method to prepare water containing extremely high concentration of H_2_ has been developed. We hypothesized that H_2_ in the water could complement conventional therapy by reducing the oxidative stress in RA.

**Methods:**

Twenty patients with rheumatoid arthritis (RA) drank 530 ml of water containing 4 to 5 ppm molecular hydrogen (high H_2_ water) every day for 4 weeks. After a 4-week wash-out period, the patients drank the high H_2_ water for another 4 weeks. Urinary 8-hydroxydeoxyguanine (8-OHdG) and disease activity (DAS28, using C-reactive protein [CRP] levels) was estimated at the end of each 4-week period.

**Results:**

Drinking high H_2_ water seems to raise the concentration of H_2_ more than the H_2_ saturated (1.6 ppm) water in vivo. Urinary 8-OHdG was significantly reduced by 14.3% (p < 0.01) on average. DAS28 also decreased from 3.83 to 3.02 (p < 0.01) during the same period. After the wash-out period, both the urinary 8-OHdG and the mean DAS28 decreased, compared to the end of the drinking period. During the second drinking period, the mean DAS28 was reduced from 2.83 to 2.26 (p < 0.01). Urinary 8-OHdG was not further reduced but remained below the baseline value. All the 5 patients with early RA (duration < 12 months) who did not show antibodies against cyclic citrullinated peptides (ACPAs) achieved remission, and 4 of them became symptom-free at the end of the study.

**Conclusions:**

The results suggest that the hydroxyl radical scavenger H_2_ effectively reduces oxidative stress in patients with this condition. The symptoms of RA were significantly improved with high H_2_ water.

## Introduction

Rheumatoid arthritis (RA) is a chronic inflammatory disease that affects approximately 1% of the population. It is characterized by irreversible joint disorder accompanied by destruction of bone and cartilage. In addition, the chronic inflammation associated with RA often damages the skin, subcutaneous tissue, and the lungs and cardiovascular system, including the pleura, pericardium, thereby diminishing the quality of life and survival time [1]. Although the etiology is unknown, RA is certainly associated with autoimmune disorders, and its pathogenesis has been well investigated [2]. Auto-reactive T cells that infiltrate the synovial tissue promote the immune response and lead to overproduction of pro-inflammatory cytokines such as TNF-α and IL6. Thus, early therapy was based on aggressive biologic modification of the disease by controlling the synovial T cells and/or suppressing the levels of the cytokines implicated in the disease.

Apart from the present immunogenic targets, reactive oxygen species (ROS) are of considerable interest. ROS is spontaneously produced as by-product during electron transfer in oxidative phosphorylation [3,4]. They are also actively produced by NADPH oxidase, which play important roles in the immune system [5,6]. ROS include superoxide, peroxide, hydroxyl radicals and reactive nitrogen species. They oxidize various cellular and extracellular components, including nucleotides, DNA, proteins, polysaccharides, and lipids, by the unpaired free radicals. The numerous products that appear to be generated by ROS have been identified in clinical samples including peripheral blood and fluid from the joints in patients with RA [7-9]. Among them, 8-hydroxyguanine (8-OHdG), which is produced by the oxidation of guanine bases in DNA and also in the nucleotide pools, is important [10-12]. 8-OHdG is a standard biomarker for oxidative stress. Numerous studies have reported that 8-OHdG accumulates in diseases related to oxidative stress, such as cancer, diabetes mellitus, Alzheimer’s disease, hypertension, chronic renal disease, cardiovascular disease, metabolic syndrome and autoimmune disease [13-18]. Elevated levels of 8-OHdG have been reported in RA [16].

Hydroxyl radical is the most toxic ROS due to the detrimental effects of its rapid and indiscriminate reactivity. Is has been shown that molecular hydrogen (H_2_) eliminates the hydroxyl radical in cultured cells and living organisms [19,20]. H_2_ does not have any influence on the other ROS, including superoxide, peroxide, and nitric oxide; these ROS play important roles in the defence system or in signal transduction [19]. In humans, the safety of H_2_ has been tested, especially in the field of deep diving; in contrast to general drugs, which usually have some harmful effects, no cytotoxicity was found even at high concentrations of H_2_[21,22]. H_2_ is an inert gas present within the human body and is not classified as a medicine, but it has been shown to have therapeutic potential for acute or chronic inflammatory diseases related to ROS [20,23]. H_2_ dissolved in water has already been used in the clinical treatment of type II diabetes mellitus [24] and metabolic syndrome [25].

At present, there is no evidence that H_2_ provides benefits for patients with RA. We might expect H_2_ to complement or provide a substitute for conventional therapy by reducing oxidative stress in RA, especially where existing drugs are insufficient in efficacy or are discontinued due to harmful side effects or due to financial considerations. H_2_ may also be useful for patients with early RA, in whom antibodies against cyclic citrullinated peptides (ACPAs) are below standard values. ACPAs are positive in about two-thirds of patients with RA and are associated with rapid joint destruction and a poor prognosis [26,27]. Patients at high risk are targeted for early aggressive immunosuppressive therapy. We therefore expected that the introduction of H_2_ into ACPA-negative patients with recent-onset RA may help rule out transient or non-aggressive RA.

In the present study, we tested the effects of drinking water containing a high concentration of hydrogen (high H_2_ water) as an anti-oxidant supplement against the hydroxyl radical, among patients with RA.

## Methods

### Patients

Twenty-two patients who fulfilled the 1987 and 2010 American College of Rheumatology criteria for RA were enrolled. All patients gave their informed consent to enroll in the study, and this study was approved by the Haradoi Hospital Ethics Committee. Two of the patients left the study during the first period. One patient did not continue drinking the water daily during winter, and the other declined to provide a blood sample every 4 weeks. The remaining 20 patients completed the study and the data are summarized in Table 1.

**Table 1:** **Clinical effects of high H**_**2 **_**water and patient characteristics at baseline**, **and 4**, **8**, **and 12 weeks**

	**Patients**		**Disease duration**		**Urinary 8-OHdG (ng/mg Cr)**				**DAS28**				**ACPA (U/ml)**				**Medications**						
					**Baseline**	**4w**	**8w**	**12w**	**Baseline**	**4w**	**8w**	**12w**	**Baseline**	**4w**	**8w**	**12w**	**MTX (mg/w)**	**Duration**		**Abatacept (mg/4w)**	**Duration**		**Response**
**Patient number**	**Sex**	**Age**	**Y**	**M**														**Y**	**M**		**Y**	**M**	
1	F	67	3	2	10.1	10.0	7.7	6.0	3.28	2.45	2.40	1.94	300	204	254	226	6	2	0	500	1	2	good
2	F	69	5	0	18.0	16.2	12.0	11.5	2.90	2.95	2.53	2.62	<0.6	<0.6	<0.6	<0.6	4	2	1				non-response
3	F	76	11	0	11.0	8.1	7.8	7.8	4.61	3.71	3.28	3.01	52.4	47.2	58.0	56.6	6	3	0				good
4	F	51	2	1	11.4	11.3	9.1	9.4	3.00	2.79	2.86	2.30	<0.6	<0.6	<0.6	<0.6	6	1	6				moderate
5	F	41	2	0	10.9	9.9	11.3	10.0	4.55	3.43	3.06	3.13	6.9	6.2	9.2	14.5	none						good
6	F	42	2	2	15.3	12.6	11.6	10.8	4.63	4.03	2.44	2.57	<0.6	<0.6	<0.6	<0.6	6	1	4				good
7	F	86	5	0	10.0	6.5	7.1	7.1	4.67	3.79	3.78	3.00	0.8	<0.6	<0.6	<0.6	none						good
8	F	40	10	0	nd	nd	3.9	4.9	2.88	1.25	1.50	0.99	13.5	14.5	15.4	16.8	6	2	2				good
9	F	41	2	1	8.7	7.7	7.3	9.0	5.38	4.35	4.37	4.22	34.3	36.4	57.6	65.7	8	1	6				moderate
10	M	55	2	3	9.3	8.3	9.7	11.5	4.62	3.44	3.28	3.06	532	258	299	223	6	1	8				good
11	F	56	2	0	12.0	8.2	10.8	10.9	5.47	5.22	5.06	4.16	<0.6	<0.6	<0.6	<0.6				500	0	8	good
12	M	40	2	1	5.2	4.9	4.9	6.4	1.95	1.96	1.52	1.59	<0.6	<0.6	<0.6	<0.6	6	1	4				non-response
13	F	64	13	0	nd	8.1	11.2	9.0	2.72	2.14	2.32	2.21	490	299	266	256	4	7	0				non-response
14	F	58	2	0	10.7	8.4	8.5	10.5	3.21	3.03	2.93	3.04	<0.6	<0.6	<0.6	<0.6	6	1	2				non-response
15	F	74	3	0	6.5	6.8	5.7	5.3	2.91	2.52	2.30	1.30	<0.6	<0.6	<0.6	<0.6	none						good
16	F	69	0	8	7.6	6.5	8.6	5.4	4.07	2.78	2.54	0.99	2	3.7	<0.6	1.9	none						good
17	M	42	0	9	7.9	5.7	6.6	7.2	3.25	2.03	2.27	1.11	<0.6	<0.6	<0.6	<0.6	6		6				good
18	F	49	0	5	10.6	8.5	7.2	9.3	4.91	3.46	2.83	1.17	<0.6	<0.6	<0.6	<0.6	none						good
19	F	57	0	10	9.5	9.4	9.2	10.7	3.38	2.87	2.50	1.82	<0.6	<0.6	<0.6	<0.6	none						good
20	F	41	0	8	5.2	5.0	4.4	3.8	4.20	2.29	2.91	0.99	<0.6	<0.6	<0.6	<0.6	none						good

And, no urine sample; DAS28, disease activity score in 28 joints; ACPA, antibodies against cyclic citrullinated peptides.

Five patients with early RA (disease duration < 12 months) who were diagnosed at our hospital were included in the study; four of them had not received any medication before the study. Patients with disease duration > 12 months had been administered disease-modifying anti-rheumatic drugs (DMARDs) and/or biological drugs. Twelve patients had been treated with methotrexate (MTX), two with abatacept (500 mg/4 w), and 1 patient with both drugs. One patient did not receive DMARDs, because she had swelling in only 1 joint for more than 2 years and occasional swelling in another joint. Two patients had discontinued therapy with MTX or biological drugs because of harmful side effects. None of the 20 patients had been treated with steroid hormones. In addition to MTX and abatacept, patients continued any other regular medication that they were on during the study.

Clinical response was measured by changes in the disease activity score in 28 joints, using C-reactive protein levels (DAS28). Remission (DAS28 < 2.3) and the decrease in the DAS28 was evaluated as good, moderate, or a non-response according to the European League against Rheumatism (EULAR) response criteria. The DAS28 was evaluated at baseline, after 4 weeks of drinking high H_2_ water, at 8 weeks after the wash-out period, and at 12 weeks after another 4 weeks of drinking the water.

### Study design

The study began in August in 2011, lasted 12 weeks and included a wash-out period. The patients drank water containing a high concentration of hydrogen (4 to 5 ppm H_2_ water) daily for 4 weeks, followed by a wash-out period of 4 weeks. They then drank the high H_2_ water again for another 4 weeks. They consumed 530 ml of the water within 1 h every day during the 4-week drinking period. The patients themselves dissolved the H_2_ in 530 ml of water each day just before drinking. They were asked to drink it as soon as possible, within a maximum of 1 h of opening the bottle. During the wash-out period, the patients did not consume any water without H_2_.

### Preparation of the high H_2_ water

Hydrogen gas was produced in an acrylic resin tube in a PET bottle to provide a carbonated drink containing 530 ml water. The amount of 530 ml fills the entire PET bottle with no space for air, which allows the highest concentration of H_2_ to be present. The material for producing molecular hydrogen was prepared by mixing 75% weight of metal aluminum grains and 25% weight of calcium hydroxide; 0.5 g of the material was enclosed and heat-sealed within a non-woven fabric. After insertion into an acrylic resin tube and the addition of 0.5 ml of water, a cap with a check valve was tightly closed. This was to prevent the drinking water from entering the tube while allowing the gas to permeate it. In about 5 min at room temperature, the material starts a reaction in the wet fabric. The reaction is as follows:

(1) $$2\text{Al}+\text{Ca}{\left(\text{OH}\right)}_{2}+{2H}_{2}O\to \text{Ca}{\left({\text{AlO}}_{2}\right)}_{2}+3\;H_{2}$$

The H_2_ gas produced is emitted into the water in the PET bottle through the check valve attached to the acrylic resin tube. During the procedures therefore, the chemical compound as well as the water for the reaction does not come into contact with the drinking water. During the reaction, the hydrogen gas lowers the surface of the water in the standing bottle, which is gradually hardened by the increasing pressure within it. After the reaction is terminated, the hydrogen gas is dissolved by shaking the bottle by decantation for about 30 s. The concentration of hydrogen gas in the water was measured according to a previously reported method [28] and verified by the apparatus, model DHD1-1 (DKK-TOA Corporation, Tokyo, Japan). The H_2_ saturated (approximately 1.6 ppm) water was produced using AQUELA BLUE instrument electrolysis (MiZ Company and ecomo International Co., Ltd) [29]. H_2_ exhaled in the breath of three independent volunteers was measured using BAS200 (Mitleben R&D Associates).

### Measurements of urinary 8-hydroxydeoxyguanine (8-OHdG) and serum ACPA

The defined marker for oxidative stress, urinary 8-OHdG, which reflects 8-hydroxyguanine in DNA, was measured according to the procedure based on the ELISA method [30]. The assay was performed at Mitsubishi Chemical Medience Inc. (Tokyo, Japan), using the test kit New 8-OHdG Check ELISA (manufactured by the Japan Institute for the Control of Aging Inc., Shizuoka, Japan). ACPAs in the serum samples were assayed at SRL Inc. (Tokyo, Japan) using the ELISA kit (MESACUP-2 test CCP; MBL Inc., Tokyo, Japan).

### Statistical analysis

One-way repeated measure analysis of variance (ANOVA) was used to identify significant differences. In repeated measures ANOVA, the sphericity assumption was always violated. In order to account for this violation, the degrees of freedom were adjusted using the Greenhouse-Geisser correction. Bonferroni’s multiple comparison was used as the post-hoc test. All data analyses were performed using SPSS Statistics 20.

## Results

**Figure 1:**
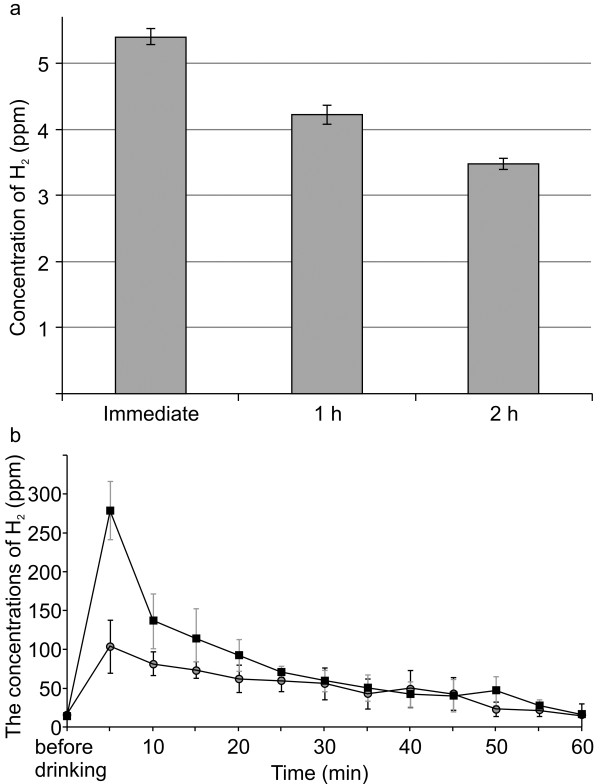
**a Concentration of H**_**2 **_**in the water at each time point. **The concentrations were measured immediately after preparation, and at 1 and 2 h after preparation. The error bars represent the mean and standard deviation (SD) for 5 independent measurements. **b** Concentration of H_2_ in the water with 1.6 ppm H_2_ (circle) or with 5 ppm H_2_ (square) at each time point. The concentrations were measured before drinking and every 5 minutes after drinking. The error bars represent the mean and standard deviation (SD) for 3 independent measurements.

As shown in Figure 1a, the concentration of H_2_ in the drinking water exceeded 5 ppm (5.40 ± 0.12 mg/L) and remained above 4 ppm (4.22 ± 0.15 mg/L) 1 h after the cap was opened. Accordingly, the amount of hydrogen in the bottle was between 2.1 mg to 2.7 mg. As shown in Figure 1b, the concentration of H_2_ exhaled in the breath before drinking the H_2_ saturated water and the high H_2_ water were 15.7 ± 2.5 ppm and 14.0 ± 4.0 ppm, respectively. These concentrations in the baseline are close to the data previously reported [31]. In 5 minutes the peak concentration was 102.7 ± 34.0 ppm and 278.3 ± 37.5 ppm, respectively. It gradually decreased in 60 minutes and returned to near baseline level, 14.0 ± 1.0 ppm and 15.3 ± 2.1 ppm, respectively. Drinking high H_2_ water raises the concentration of H_2_ more than the conventional H_2_ saturated water in vivo.

During the drinking period, patients usually passed urine 1 or 2 times more than usual, as they drank the extra water. No other adverse effects were observed.

**Figure 2:**
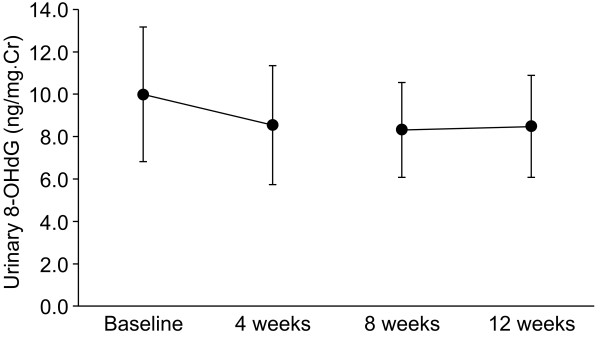
**Urinary 8**-**OHdG in 18 patients measured at baseline and at 4**, **8 and 12 weeks. **Error bars represent the mean and standard deviation (SD) for measurements in 18 patients.

Urinary 8-OHdG was analyzed in 18 patients (Table 1). During the first 4 weeks, 8-OHdG decreased in 17 of these patients after drinking the high H_2_ water. As shown in Figure 2, the mean decrease in 8-OHdG among the 18 patients was 14.3% (9.99 to 8.56 ng/mg Cr, p < 0.01). Drinking the high H_2_ water for 4 weeks affected the urinary 8-OHdG for 4 weeks after the wash-out period, with urinary 8-OHdG remaining below the baseline level at the end of the break (mean, 8.31 ng/mg Cr). The patients did not drink extra water during this period and the frequency of urination did not increase. At 12 weeks, urinary 8-OHdG had decreased by 15.1% (9.99 to 8.48 ng/mg Cr, p < 0.01). Nine of the eighteen patients had decreased urinary 8-OHdG in both drinking periods. There was no increase in urinary 8-OHdG in either drinking period.

**Figure 3:**
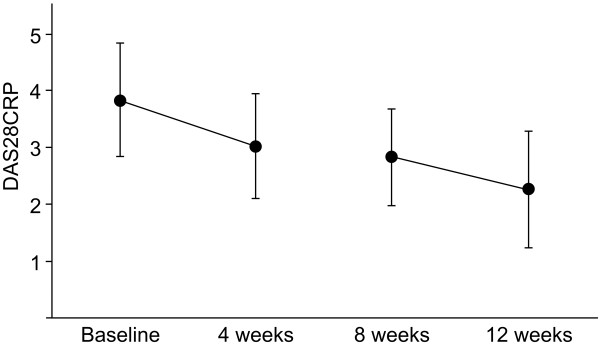
**DAS28 scores at baseline**, **and 4**, **8 and 12 weeks. **Error bars represent the mean and standard deviation (SD) for measurements in 20 patients.

The DAS28 results are shown in Table 1. The scores decreased in 18 of the 20 patients during the first drinking period, with a decrease more than 0.6 points in 12 of the patients, indicating a significant improvement in the symptoms of RA. Moderate improvement (0.6 < DAS28 ≤ 1.2) was observed in 6 patients and good improvement (DAS28 >1.2) in 6 other patients. After the wash-out period, the DAS28 remained better than that at the baseline in all 20 patients. In 14 of the 20 patients, the scores remained below the level immediately after the first drinking period. At the end of the study, 16 patients showed significant improvement in the DAS28. Two of these had a moderate response and fourteen had a good response. Nine patients achieved remission (DAS28 < 2.3), except for one patient who had been in remission at the baseline assessment. Although all 20 patients had decreased disease activity at the end of the study, 4 patients did not experience improvement in symptoms (DAS28 ≤ 0.6). On average, the score decreased from 3.83 to 3.02 (p < 0.01) in the first drinking period and from 2.83 to 2.26 (p < 0.01) in the second drinking period (Figure 3). During the wash-out period, the DAS28 remained below the baseline, and decreased from 3.02 to 2.83.

Five patients (numbers 16 to 20 in Table 1) had been classified as having early RA (duration < 12 months) and were ACPA-negative. Four of them had not taken any medications for RA before the study. The patients’ symptoms improved by more than 0.6 points in the first drinking period. At the end of the study, their DAS28 scores were all below 2.3, indicating that remission was achieved. Four of the patients did not report any swollen or painful joints and were symptom-free.

Seven of the 20 patients were ACPA-positive (above 4.9 U/ml) at each time point (Table 1). The ACPA levels were below 100 U/ml in 4 patients at baseline and did not decrease during the study; the levels showed slight increase in three patients. The disease activity, however, improved in the 4 patients. Three of them showed a good response and one of them a moderate response. On the other hand, in three patients with ACPA above 300 U/ml, there was a mean decrease from 441 U/ml to 235 U/ml. The response was diverse among the patients, with a good response observed in 2 patients and no response in another. Although the direct effect of high H_2_ on ACPA is unclear at present, 5 of the 7 ACPA-positive patients showed a good response to high H_2_ water, as assessed by improvement in the DAS28.

## Discussion

Many studies have demonstrated that ROS play some noteworthy roles in the pathogenesis of RA, and consequently, they have become therapeutic targets in this condition. The ROS scavenger superoxide dismutase (SOD) was expected to have clinical efficacy, and initially, bovine SOD was injected into the joints of patients with RA [32]. However, in relation to the exogenous SOD protein, results from clinical trials have not been reproduced. Simulators of SOD, which have been shown to have considerable anti-inflammatory effects in animal models of arthritis, are still being investigated [33,34]. Edaravone, which is approved for the treatment of acute ischemic stroke, is another ROS scavenger [35]. A clinical report describes treatment of a patient with RA and ischemic stroke with edaravone, and raises the possibility of further clinical trials [36], but the results do not establish whether ROS scavengers are efficient in reducing the disease activity in patients with RA. Direct evidence is also lacking about whether ROS prime the autoimmune response or reduce chronic inflammation in RA.

Molecular hydrogen stands out as an antioxidant due to its selectivity for the hydroxyl radical and its permeability [19]. Its physical properties allow it to diffuse through the cellular membranes and rapidly leave the body. Due to these properties of H_2_ we expected reduction in ROS in RA patients with a better efficacy. In our study, patients who drank the high H_2_ water had marked reduction in disease activity, and a significant decrease in urinary 8-OHdG.

It has been demonstrated that molecular hydrogen ameliorates inflammation by down-regulating TNF-α [20,37]. It is known that ROS including hydroxyl radicals function as second messengers for the production of TNF-α [38,39]. There is a possibility that the anti-inflammatory properties by H_2_ may be through inhibition of the TNF-α pathway. It has been reported that hydrogen-rich saline prevented neointima formation by suppressing the NF-kB and TNF-α cascade which is involved in the redox-sensitive mechanisms in immune system [40]. The reduction of 8-OHdG and the disease activity in RA patients presented here could be the results of downregulation of TNF-α pathway through scavenging hydroxyl radicals which occur upstream of the inflammatory cascade.

It should be noted that the influence of H_2_ on the disease activities and oxidative stress was sustained even during the wash-out period. These continuous anti-inflammatory effects of H_2_ are observed with both the ACPA-negative and the ACPA-positive patients who were treated with immunosuppressive medications. The marker for oxidative stress, 8-hydroxyguanine is highly mutagenic because it pairs with adenine as well as cytosine. These properties cause partial phenotypic suppression during transcription as well as transversion mutations during DNA replication [41]. It is likely that the error proteins, produced as a result of the altered genetic information, therefore, are identified as foreign molecules and play a role as neo-epitopes [42,43]. They would then continue to activate the immune system. The sustained effects of H_2_ during the wash out period may reflect the decrease of such neo-epitope. On the other hand, there was no significant decrease of DAS28 during the washout period. It may indicate that the pathogenesis of RA did not disappear completely at the end of the first drinking period. It is necessary to investigate the transition of pro-inflammatory cytokines including TNF-α, both during the drinking period and wash out period for further studies.

The prognosis of RA has improved during this decade, and a so-called paradigm shift has been achieved using early aggressive therapy to biologically modify the disease [44,45]. Remission is not difficult to achieve; however, it is difficult to determine the role of drugs in remission [2]. It is difficult to determine at which stage patients will be able to discontinue medication without recurrence of inflammation, and sustained medication is often necessary. Most of these problems are attributed to the intense immunosuppressive procedures, and the epitopes involved are still unknown. Patients with RA are classified according to their prognosis, as having progressive RA, which requires early aggressive disease-modifying biologic therapy, or as having transient RA, which may show spontaneous remission. It is difficult to distinguish transient from progressive RA in the early stages [46].

Although ACPAs have been useful for diagnosing RA, they are less sensitive (48%) in early RA [26]. In the present study, the 5 patients with early onset and ACPA-negative RA all achieved remission after drinking the high H_2_ water; possibly, some of them had transient RA.

In total 47.4% of patients (9/19) achieved the remission, except for one patient who had been in remission at the baseline assessment. Although the efficacy of H_2_ presented here is not inferior to those in recently reported studies on tocilizumab [47], adalimumab plus MTX [48] or MTX monotherapy [49] , the study here is non-controlled, on a smaller scale and also, the patient background and the period of medication varied. Estimation of the efficacy of high H_2_ water in RA patients should be done carefully in a further placebo-controlled study. The high H_2_ water was produced in a bottle by using a novel high-pressure method. Since molecular hydrogen is a very small molecule and is easily lost even through the wall of PET bottles with the passage of time, the patients had to prepare it themselves every day. Also, H_2_ is an inert gas having no taste or smell. It was difficult to set up placebo controls using another inert gas and a similar preparation. However, it is important to include placebo controls in future studies.

## Conclusion

We suggest that high H_2_ water may be useful to complement conventional RA therapy by reducing oxidative stress, especially in early stage and ACPA-negative RA, to assist diagnosis and treatment decisions. Further study is required to confirm this theory. However, high H_2_ water is freely available, and its benefits could also be demonstrated spontaneously by observing the disease rates in people who regularly drink it.

## Abbreviations

RA: Rheumatoid arthritis; ROS: Reactive oxygen species; ACPA: Antibodies against cyclic citrullinated peptide; DMARD: Disease-modifying anti-rheumatic drug; MTX: Methotrexate; DAS28: Disease activity score in 28 joints; 8-OHdG: 8-hydroxydeoxyguanine; SOD: Superoxide dismutase.

## Competing interests

The authors declare that they have no competing interests.

## Authors’ contributions

TI designed the study and estimated all of the data. Also, he formulated and tested the hypotheses and derived conclusions. BS, TS, and RK developed and prepared the materials for high H_2_ water. They also collected the data for the concentration of H_2_ in the water and breath. MR showed patients how to prepare the high H_2_ water and collected data. YH, YN, and HH supported this study by offering space, collecting data, and giving advice. T. Nagao helped in designing this study and gave advice on many aspects. All authors read and approved the final manuscript.
